# Acute glans ischemia after circumcision successfully treated with low-molecular-weight heparin and topical dihydrotestosterone: A case report

**DOI:** 10.1097/MD.0000000000021340

**Published:** 2020-07-17

**Authors:** Zlatan Zvizdic, Dusko Anic, Nusret Popovic, Semir Vranic

**Affiliations:** a Clinic of Pediatric Surgery; b Pediatric Clinic, University Clinical Center Sarajevo, Sarajevo, Bosnia and Herzegovina; c College of Medicine, QU Health, Qatar University, Doha, Qatar.

**Keywords:** circumcision, complications, glans ischemia, treatment

## Abstract

**Rationale:**

Circumcision like any other surgical procedure is not devoid of complications. Serious complications are rare and include iatrogenic hypospadias, glans ischemia/necrosis, and glans amputation, all of which require an emergent treatment.

**Patient concerns:**

We report here a case of 6 months-old-boy with a superficial glans ischemia following circumcision.

**Diagnosis:**

Physical examination revealed a severely cyanotic glans with the moderate edema of the dorsal penile skin. Plasma levels of D-dimer were 8.57 mg/L. Urine passage was unremarkable while color Doppler ultrasonography revealed a normal blood flow.

**Interventions:**

The patient was successfully treated with subcutaneous injection of enoxaparin (low-molecular-weight heparin) and topical 2.5% dihydrotestosterone.

**Outcomes:**

The appearance of the glans penis on the 5th day was close to normal while the control levels of D-dimer dropped to the reference range. The patient was discharged from the hospital on the 6th day. At 6-month follow-up, the appearance of the glans penis was normal.

**Lessons:**

Acute glans penis ischemia following circumcision is a rare complication. Its successful treatment with enoxaparin and topical dihydrotestosterone has not been previously reported in the literature.

## 1 Introduction

Circumcision is one of the most commonly performed surgical procedures in clinical practice.^[1]^ Although a simple surgical procedure, circumcision is not completely devoid of complications (frequency 2–10%).^[2]^ These include bleeding, wound infection, glans amputation, and structural deformities. Among the complications, ischemia or even necrosis of the glans penis is one of the rarest.^[3]^ The causes of ischemia of the glans penis may be due to vasoconstrictors applied during local anesthesia, arterial vasospasm due to a needle microtrauma during dorsal nerve block, blood vessel binding, excessive use of monopolar electrosurgery, tight suture line, and/or tight bandage applied to the circumcised area.^[4]^

There are no uniform treatment guidelines at present. The recommended approaches include hyperbaric therapy (HBOT), topical 10% testosterone undecanoate, intravenous, or oral pentoxifylline (PTX), low-molecular-weight heparin (enoxaparin), intracavernous glycerol trinitrate and bupivacaine, intravenous infusion of iloprost, antiplatelet, corticosteroids, and peridural anesthesia.^[4–6]^

We report here a rare case of 6 months-old-boy who presented with a superficial glans ischemia following circumcision that was successfully treated by a combination of a subcutaneous injection of enoxaparin with topical 2.5% dihydrotestosterone (DHT).

## 2 Case report

A 6-months-old boy was admitted to our department with discoloration of the glans penis. The boy was subjected to the circumcision under local anesthesia (2% lidocaine without adrenaline) a day earlier at another institution. *Medical history indicated that that discoloration of the glans had started several hours after the circumcision and gradually progressed.*

**Figure 1:**
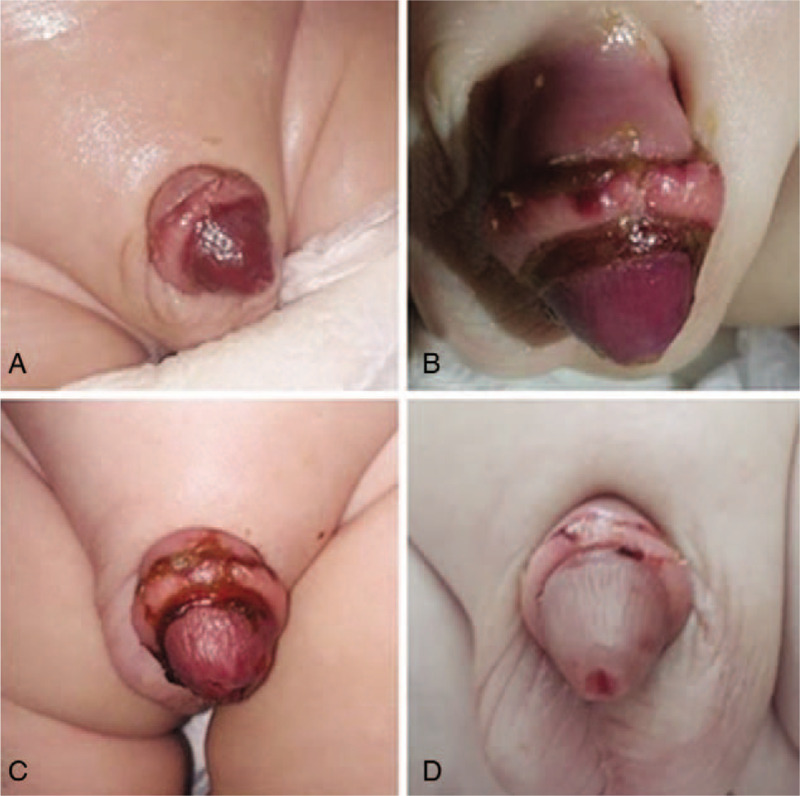
(A–D) A gross appearance of the glans penis on the second day (A), third day (B), fourth day (C), and fifth day (D) following the treatment with enoxaparin (low-molecular-weight heparin) and a topical 2.5% dihydrotestosterone.

Physical examination revealed a severely cyanotic glans with a moderate edema of the dorsal penile skin (Fig. 1A). Plasma level of D-dimer was 8.57 mg/L (normal level 0–0.5 mg/L). Urine passage was normal. Color Doppler (CD) ultrasonography revealed a normal blood flow. A diagnosis of superficial glans ischemia was proposed. A conservative treatment started immediately with a subcutaneous injection of enoxaparin as a single daily dose of 1.25 mg/kg and topical 2.5% DHT twice daily. Two days after the onset of treatment, the glans penis became less livid hue (Fig. 1B and C). The appearance of the glans penis on the 5th day was close to normal (Fig. 1D) while the control levels of D-dimer dropped to the reference range. The treatment with enoxaparin and DHT was consequently discontinued. The patient was discharged from the hospital on the 6th day. At 6-month follow-up, the appearance of the glans penis was normal.

## 3 Discussion

Circumcision is a common surgical procedure that is carried out for medical, religious, and cultural reasons. Although simple, it is not devoid of complications.^[2–4]^ Most of these complications are minor and easily treatable. Serious complications (e.g., ischemia and necrosis of the glans penis) are rare, but well described in the literature.^[4–7]^ The etiology of ischemia of the glans after circumcision remains however unclear. We believe that in our case ischemia might be due to the tight bandage on the base of glans with a consequent venous obstruction. The outcome of the complications ranges from transient superficial glans ischemia to glans necrosis and glans amputation with a risk of future meatal stenosis and urethral stricture.^[8]^ Untreated ischemia of the glans can lead to irreversible necrosis with the possible consequence of loss of the entire glans.

Glans ischemia has been sporadically reported in the literature only as case reports with proposed modalities of treatment that included HBOT, PTX, enoxaparin, iloprost, antiplatelet, corticosteroids, topical testosterone, and peridural anesthesia.^[4,5,9]^ Enoxaparin has been shown to be a safe anticoagulant *in primary prophylaxis and treatment of thromboembolism* in children.^[10]^*Efe et al suggested that the glans ischemia might be successfully treated with enoxaparin,* especially in cases when the D-dimer levels are increased.^[6]^ In our case, *D*-*dimer* levels were markedly *elevated* and an administration of enoxaparin was a rational therapeutic approach.

Several experimental studies have explored the effects of testosterones on vascularity. Franck-Lissbrant et al found that testosterone treatment restores the endothelial proliferation rate and blood vessel weight in castrated rats.^[11]^ Stern et al demonstrated that the testosterone treatment of human foreskin in a transplant model increased neovascularization and decreased fibrosis.^[12]^ There is also an evidence that topical 10% testosterone application may have beneficial effects on the ischemic/necrotic glans leading to its complete recovery.^[12]^ Based on in vitro studies, Aminsharifi et al hypothesized that the testosterone treatment affected the endothelial cells and vascular endothelial growth factor (VEGF) expression with a consequent increase in the penile blood supply and revascularization of the glans.^[9]^ In our case, the boy was treated with enoxaparin therapy in association with topical 2.5% DHT. To the best of our knowledge, the use of such a combined treatment has not been described previously.

In conclusion, we demonstrated that the glans ischemia after circumcision can be successfully treated by a combined injection of enoxaparin and topical 2.5% DHT leading to a complete recovery of the glans penis.

## Author contributions

**Conceptualization:** Zlatan Zvizdic, Semir Vranic.

**Data curation:** Zlatan Zvizdic, Dusko Anic, Nusret Popovic.

**Formal analysis:** Zlatan Zvizdic, Semir Vranic.

**Investigation:** Zlatan Zvizdic, Dusko Anic, Nusret Popovic.

**Resources:** Semir Vranic.

**Writing – original draft:** Zlatan Zvizdic, Dusko Anic, Nusret Popovic, Semir Vranic.
